# Well-Being amongst College Students during COVID-19 Pandemic: Evidence from a Developing Country

**DOI:** 10.3390/ijerph192416745

**Published:** 2022-12-13

**Authors:** Lina Martinez, Lina Sofia Valenzuela, Victoria Eugenia Soto

**Affiliations:** 1 Business School, Universidad Icesi & POLIS, Cali 760031, Colombia; 2 Entrepreneurship Development Center, CDEE, Universidad Icesi, Cali 760031, Colombia; 3 PROESA, Universidad Icesi, Cali 760031, Colombia

**Keywords:** well-being, COVID-19, college students, gratitude, optimism

## Abstract

College students face unique challenges that the consequences of COVID-19 might aggravate. To explore the pandemic’s consequences on college students’ well-being, we conducted an online survey with 634 students from a private university in Cali, Colombia. The study sought to assess students’ well-being due to COVID-19, and to explore the mediating effects of optimism, gratitude, and emotional closeness on college students’ well-being. Results showed that COVID-19 affected students’ mental health and well-being. Being optimistic and grateful mediated with life satisfaction and happiness. Optimism, emotional closeness, and gratitude also mediated the negative effect of fear of infection and the pandemic’s impact on students’ academic performance. The results of this analysis will promote discussion of the implementation of coping strategies to help students thrive, promote resilience, and contribute to students’ well-being and better mental health.

## 1. Introduction

The restrictions associated with the containment of COVID-19 are, without a doubt, the “largest physiological experiment even conducted” [1]. With over two million deaths worldwide and more than 100 million confirmed cases [2] the pandemic, the measures taken to control the spread of the virus, and the global economic consequences, are imposing a heavy toll on our mental health. This crisis and the pervasiveness of feelings such as fear and uncertainty have affected the entire global population to a viable extent.

Quarantine, the most used measure to contain the pandemic contagious rate, has a negative psychological effect: producing stress, confusion, and anger. It also adds stressors, such as fear, frustration, boredom, and financial concerns [3]. For some population groups, such as children and young adults, the pandemic may have more severe consequences on mental health, even more significant than the consequences on their physical health [2].

College students face unique challenges that negatively affect their mental health. Before COVID-19 unfolded, there were reports of the prevalence of poor mental health worldwide amongst this population. The rates of anxiety and depression are steadily increasing. Information from the US indicates a spike of 63% of young adults (18 years and over) reporting symptoms associated with significant depression between 2005 and 2017, mainly affecting girls [4]. Worldwide, one in five college students experiences one or more diagnoses of mental disorders [5].

College students can be subject to stressful situations. The pressures of establishing a career path, academic demands, the transition from adolescence to adulthood, peer pressures, and many other factors affect their general well-being and mental health [6]. Before the pandemic, there was a significant number of reports showing that the increase in stress, depression, and reduction in the overall well-being amongst college students was prevalent, and was the subject of different interventions from the public health system and colleges initiatives [7,8,9]. The current pandemic represents an additional challenge for this population. Changes in the routine, the reduction of personal interactions with classmates and friends, and the interruption of academic and professional trajectories negatively impact their well-being [5]. Moreover, college students around the world have presented increased stress and anxiety levels [10,11,12,13], and this may be exacerbated in the long run by uncertainties and the increase in information flow [14].

Most of the literature regarding subjective well-being amongst college students comes from developed countries [15] and explores two broad themes: on the one hand, there is a bulk of research explaining and quantifying the prevalence of stress, anxiety, depression, and worry; on the other hand, studies focused on life satisfaction, and overall well-being encompass the study of mediators that increase life satisfaction. The studies focusing on the prevalence of negative emotions show the substantial prevalence of stress, anxiety, depression, and worry amongst college students [6,7,8,9]. Generally speaking, the literature shows that young people (12 to 25 years) present a higher prevalence of negative emotions that are detrimental to overall well-being [16,17,18,19]. The group of studies focusing on life satisfaction explores positive psychological characteristics of students’ well-being. Amongst these studies, positive attributes, such as self-esteem, social support, gratitude, and optimism, amongst other copying strategies, are studied as mediators. Overall, these analyses show that positive psychological characteristics are strongly associated with students’ life satisfaction [20,21,22,23].

This analysis falls into the literature that explores well-being, its mediators, and coping mechanisms. The literature shows that coping strategies (response to a threat or emotion that exceeds the person’s resources [24], such as a positive outlook during stressful situations (optimism), being grateful, and having close relationships, can be pivotal to overcoming adverse circumstances.

### 1.1. Optimism

Optimism is defined as an attitude associated with expectations of the future, which individuals regard as positive to his/her advantage [25]. Optimism is fundamental in times of uncertainty because optimistic people tend to cope better with difficult situations [26]. Dispositional optimism helps people to cope with stress because optimistic people manage, in a better way, stressful and challenging events, accepting them instead of trying to wish them away [27]. Optimistic people also try to actively resolve problems compared to pessimists that tend to avoid coping strategies [28,29,30,31]. Among college students, optimistic individuals are more committed to social, academic, and personal activities, and positive psychological functioning [32]. Optimism can help college students to adjust in a better way during stressful situations [33], reduces feelings of loneliness [32], and is a predictor of life satisfaction and positive emotions [34]. It has also been found that students with higher levels of optimism have higher retention rates and higher grade point averages because they have higher levels of motivation and are better at adjusting and adapting to stressful situations [27,35]. Optimism contributes to subjective well-being, and college students with high optimism tend to experience positive emotions and higher life satisfaction [36,37]. Optimism also proved to be pivotal during the lockdown for college students. Evidence from Spain shows that optimism moderated educational achievement and improved grades during the pandemic for the population analyzed [38].

### 1.2. Gratitude

Most authors define gratitude as a state where grateful individuals have a high level of appreciation for life and a subjective feeling of thankfulness [36,37]. Gratitude is also defined as a trait that can vary among individuals [22,39]. Wood [40] suggested that gratitude should be conceptualized as a life orientation, where grateful people appreciate the world’s positive things. Gratitude is an emotion that is felt towards another person or thing, and it is a disposition to express and feel the emotion of thankfulness across situations [36]. Gratitude brings positive emotional states [41], is related to prosocial behavior [42], satisfaction and acceptance of difficult circumstances [43], happiness [44], and long-term subjective well-being [39,40]. In college students, being grateful contributes to their happiness [45], improves retention and academic performance [46], and promotes social support [47]. In the context of the pandemic, the evidence available shows that gratitude lessened mental health difficulties and promoted resilience [48], and that interventions fostering gratitude were effective in improving the mental health of college students during the crisis [49].

### 1.3. Emotional Closeness—Relationships

The presence and quality of supportive relationships are necessary for every aspect of the life of any individual. It is crucial to build relationships with people who can offer advice, help, or assistance during different life cycle episodes [50]. Supportive relationships facilitate coping [24], moderate the stress–depression relationship [51], and predicts a reduction in loneliness [52]. Relations with family, friends, and romantic partners are related to subjective well-being [53]. Among college students, those who spent time with family and friends have higher perceived happiness levels [54]. Social support and social connections help students adjust better to college [55] also relate to positively perceived happiness and well-being [56,57]. Emotional closeness and social connectedness were pivotal for wellbeing during the pandemic, providing a strong sense of being in control and improving emotional well-being [58]. In the higher education context, strategies aimed at grouping students showed that group support provided opportunities for improving academic achievement and outlets for emotional support [59].

This study aimed to contribute to the bulk of the literature focusing on students’ well-being and life satisfaction by analyzing the mediating effect of optimism, gratitude, and emotional closeness, on well-being during the pandemic caused by COVID-19. Additionally, this study contributes to the literature by providing evidence from a developed country (Colombia), where information about college students’ well-being and the pandemic are scarce. This analysis is composed of six sections, including this introduction. Section 2 discusses the analytical framework and research questions that guide our analysis. In the Section 3, we present the dataset’s details to answer the research questions and the methods. The Section 4 presents the results and ends with the discussion and conclusions of this research.

## 2. Analytical Framework and Hypothesis

**Figure 1:**
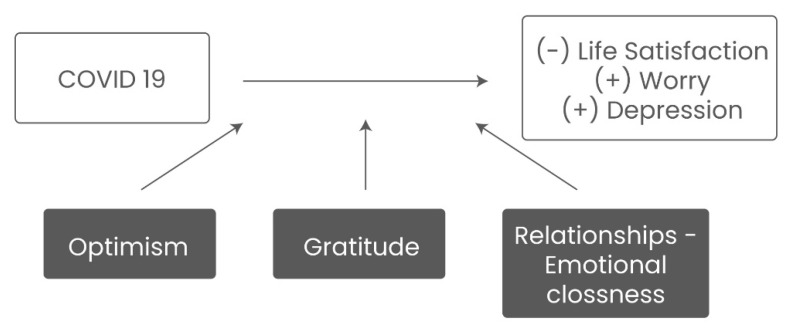
Analytical framework.

Our analytical framework assumes that COVID-19 represents a stressful event for college students, which reduces their overall well-being (life satisfaction) and increases the prevalence of emotions such as worry and depression. However, coping strategies such as a positive attitude, being grateful, and having close relationships with peers and family can mediate college students’ negative emotional states during the current crisis. Figure 1 presents the analytical framework guiding this study.

### Research Questions and Aims

Coping strategies such as practicing gratitude, staying close to loved ones and being optimistic exert protective effects against worry and depression. The literature’s significant bulk shows a positive correlation between those coping approaches and better mental health [60]. This research aimed to evaluate college students’ coping strategies to assess how those strategies help them cope during confinement.

This analysis has two purposes: first, to assess students’ well-being and mental health during COVID- 19; second, to explore the mediating effects of optimism, gratitude, and supportive relationships on college students’ well-being and mental health. Three hypotheses guided our analysis:

**Hypothesis** **1** **(H1):**  Some of the negative consequences of COVID-19 on students’ psychological well-being will be attenuated for grateful students insofar as grateful students cope better with the aftermath of COVID-19.

**Hypothesis** **2** **(H2):**  Some of the negative consequences of COVID-19 on students’ psychological well-being will be attenuated for optimistic students because optimistic students tend to have a positive outlook towards difficult circumstances.

**Hypothesis** **3** **(H3):**  Some of the negative consequences of COVID-19 on students’ psychological well-being will be attenuated by closer relationships with parents and friends, since students who have at their disposal supportive relationships are better at coping with stress and difficulties.

To answer these questions, we conducted an online survey in April 2020, after one month of quarantine in Colombia. The respondents were 634 students from a private university in Cali, Colombia. The study asked students about their general well-being and the prevalence of feelings of worry and depression. The survey also inquired about students’ attitudes toward optimism and gratefulness.

## 3. Data and Methods

**Figure 2:**
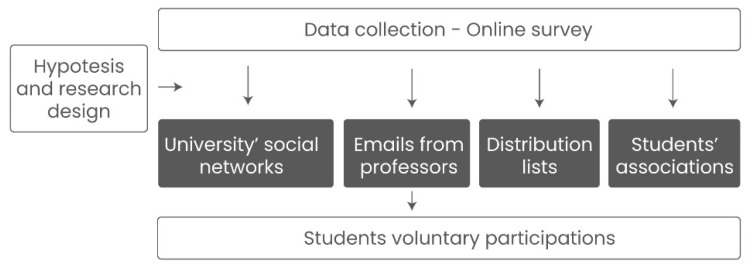
Study design.

This analysis was derived from an online survey conducted between mid-April and early May of 2020, one month after the quarantine unfolded in Colombia. The survey was uploaded into “Typeform,” a web server for online polling and surveys. For distributing the survey, researchers used a convenience sampling strategy and disseminated the online survey through four channels: (i) the university’s social networks; (ii) emails sent from professors to their students; (iii) distribution lists; and (iv) students’ associations. Survey participation was voluntary, and before starting the survey, the researchers provided a complete description of the research aims and data use, students participating consented to use the aggregated data for academic purposes. The survey was anonymous, and no personal information was collected to ensure complete anonymity. A total of 19% of students participating in the study were 16 or 17 years old (minors under Colombian legislation) at the moment of the survey. However, given the anonymous nature of the survey and their consent, we included their responses in the analysis. In total, 634 students between 16 and 24 completed the survey, 10% of the university’s total undergrad population, making a satisfactory rate compared to the median web survey participation [61]. At the survey time, students had online synchronous classes, most living at their parent’s homes. Their classes had the same content as face-to-face classes. One significant change was that the final evaluation was qualitative: ‘approve or not approved’ instead of numeric. The survey competition took about ten minutes, and before the final questionnaire was released, the survey was piloted with 20 students. The ethics committee of Universidad Icesi approved the study (code # 278). Figure 2 presents the study design.

After survey competition, students downloaded a gratitude journal and a stress management diary designed for this study at the end of the survey as a reward for participation. We included the gratitude journal as a reward for participation due to the evidence indicating that writing about gratitude has a positive effect on immediate thoughts of participants [62].

The survey inquired about subjective well-being, mental health, and concerns about the consequences of COVID-19, optimism, gratitude, and emotional closeness with parents and friends. In total, the survey had 17 questions. Appendix A presents the questionnaire, and data is available with complete description of the metrics used at Mendely data repository [63].

### 3.1. Study Variables

#### 3.1.1. Well-Being and Mental Health

In this study, we adhered to the World Health Organization’s overall concept of mental health. This idea refers to mental health as a state of well-being in which individuals can cope with the stress of life and is not restricted to the diagnosis of a mental disorder [2]. To measure well-being and the prevalence of the most common negative emotions affecting mental health (worry and depression), we used the standardized and validated scale of core well-being measures [64].

Well-being is measured using subjective and experienced well-being variables. Measures of subjective well-being come from self-reported life satisfaction. We asked students, “how satisfied are you with all aspects of your life? Answers ranged from 0 to 10, with 0 referring to completely dissatisfied and a 10 to completely satisfied.

The estimation of experienced well-being comes from using one positive emotional state: happiness and two negative states: worried and depression that students experienced the day before of the survey. The following questions were asked: Overall, how happy did you feel yesterday? How worried did you feel yesterday? And how depressed did you feel yesterday? Students’ answers were also reported on a scale from 0 to 10, with 0 the lowest and ten the highest [64].

#### 3.1.2. Concerns and Feelings about COVID-19 Consequences

The study inquired about the economic, health, and academic consequences of the coronavirus and the government’s perception of social distancing measures. We used three questions in which students were asked about how much they agreed or disagreed, measured on a 0–10 scale:

- I am concerned about the financial consequences of the coronavirus;
- The probability of that a family member or I, acquire the virus is high;
- I am concerned that my academic performance will be affected by the coronavirus.

#### 3.1.3. Optimism

The survey included a short version of the originally ten item-long Life Orientation Test-Revised (LOT-R). Students were asked: (i) In uncertain times, I usually expect the best; (ii) I am always optimistic about my future; and (iii) Overall, I expect more good things to happen to me than bad. The score was on a scale from 0 (completely disagree) to 10 (completely agree). The Cronbach reliability for the scale was 0.82 (Table 1), similar to the Cronbach alpha of the original inventory 0.82 [28]. The mean of the score of these three items was the measure of students’ optimism.

#### 3.1.4. Gratitude

Measures for gratitude come from a short version of a gratitude self-reported questionnaire, validated to Spanish [65]. The questions assessed individual differences in experiencing gratitude in daily life. Two items (3 and 5) in this scale had reversed scores. This measure included the following items:

- I have so much in life to be thankful;
- If I had to list everything that I felt grateful for, it would be a very long list;
- When I look at the world, I don’t see much to be grateful;
- I am grateful to a wide variety of people;
- Large amounts of time can go by before I feel grateful for something or someone.

Students’ responses ranged from 0 to 10. The reliability of gratitude items was 0.90, showing good internal consistency for the instrument (Table 1).

#### 3.1.5. Emotional Closeness—Relationships

The survey asked students to score from 0 to 10 “how emotionally close do you feel towards (1) your parent(s)/legal guardian(s) and (2) your friends; your significant other, and your college classmates. For this analysis, we defined two dichotomic variables: emotional closeness to parents; and emotional closes to friends.

### 3.2. Mediation Analysis

**Figure 3:**
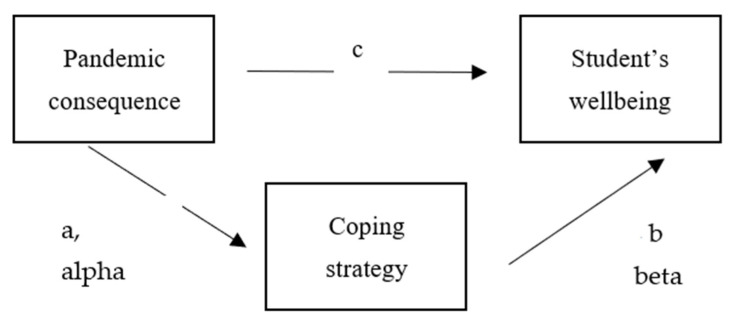
Mediation analysis of coping strategy on students’ wellbeing.

For analysis, we used a modifying Baron and Kenny’s [66] approach to establish the mediation of each coping strategy on students’ wellbeing. Following Mehmetoglu [67], the mediation effect was estimated following these steps. First, we fit a structural equation model (SEM) of coping strategy on students’ wellbeing by controlling for pandemic consequence to estimate simultaneously direct (c) and indirect paths (a alpha, b beta) (Figure 3). If either one path was not significant (or both were not significant) there was no mediation. Evidence of mediation was provided when both Pandemic Consequences → Coping Strategy and Coping Strategy → Wellbeing outcome coefficients were statistically significant. After that, Sobel’s z test was calculated to estimate the relative sizes of the indirect (mediated) vs. direct paths. The results of no, partial or complete mediation were determined as follows: (i) if the Sobel’s z test was significant and the direct path of Pandemic consequence → Wellbeing outcome was not, the mediation was full or complete; (ii) If both the z and the direct path of Pandemic consequence → Wellbeing outcome were significant, the mediation was partial; (iii) If the z was not significant but the direct path of Pandemic consequence → Wellbeing outcome was, the mediation was partial in the presence of a direct effect, (iv) If neither the z nor the direct path of Pandemic Consequence → Wellbeing outcome were significant, the mediation was partial in the absence of a direct effect. We also tested the coping strategy mediation hypothesis by using 634 Monte Carlo replications. Additional explanations of the testing mediation hypothesis can be found in Mehmetoglu [67].

For modeling, we used a medsem package in Stata 15 [67], which employs structural equation modeling to estimate the statistically significance of each path, controlling for age and gender. Coefficients were standardized. The effect of size of indirect effect of each coping strategy was estimated by the ratio of the indirect effect to the total effect ($RIT=\frac{a*b}{\left(a*b\right)+c}$) and the ratio of the indirect effect to the direct effect ($RID=\frac{a*b}{c}$).

## 4. Results

Students who participated in the study were, on average, 19.4 years old (std = 2.0), and 56% of the responders were female. To the question about how satisfied you with all aspects of your life are, students reported on average 6.8 (std = 1.8). Although it is not strictly comparable, yearly life satisfaction measures in Cali report constant life satisfaction scores of 8.5, almost two units above students’ life satisfaction scores during the quarantine. This score of 8.5 has not changed, importantly, between 2014 and 2019 and is the same as national statistics [68].

**Figure 4:**
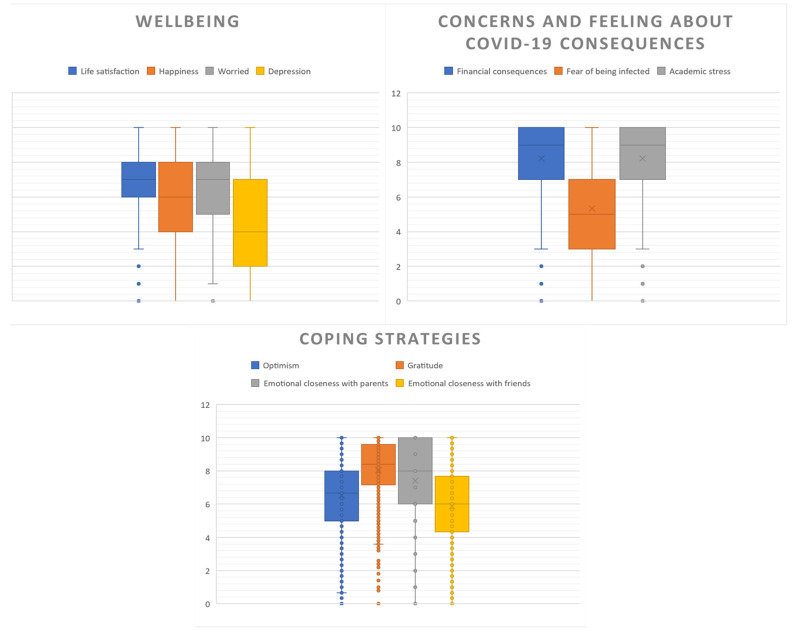
Well-being scales, COVID-19 pandemic’s concerns and coping strategies.

Students reported being happy the day before on 5.9 (std = 2.3). This number contrasted with being worried, which scored high among students, 6.4 (std = 2.6) on average. Being depressed scored 4.5 (std = 2.9). As expected, correlations between life satisfaction and happiness were positive, and negative with feelings of worry and depression (Figure 4).

Taken all together, students were highly optimistic and grateful. Gratitude was the coping strategy with the highest score (7.8, std = 2.6). These strategies were correlated among them, suggesting that these measures were related to similar feelings that enable people to overcome unpleasant situations. In contrast, optimism and gratitude correlated positively with well-being variables. Table 2 presents the correlation scores for the variables of this study.

### The Mediating Effect of the Coping Strategies

Thes mediation effect of coping strategies was mainly observed for the relationship between the financial concerns raised by COVID-19 and students’ life satisfaction (Table 3). Table 4 present the results of mediation analysis of coping strategies on positive and negative students´ subjective well-being outcomes. About 197% (RIT) of this relationship was mediated by optimism, 368% by gratitude, 213% by emotional closeness to parents and 230% by emotional closeness to friends. The ratio of the indirect effect of the coping strategy on the effect of financial consequences of COVID-19 (RID) was higher than 100%.

Regarding the mediating effect (Table 4) of the coping strategies in the relationship of life satisfaction and the fear of being infected by coronavirus, optimism and emotional closeness to friends turned out to be statistically significant. Gratitude played a partial mediating effect in this relationship, and emotional closeness to parents does not have a mediating effect at all. Concerning the mediating role of coping strategies in the relationship of the stress affecting student academic performance and life satisfaction, none of the coping strategies played a role in this relationship.

Turning to the relationship of financial pandemic consequences and happiness, optimism, and emotional closeness to parents and friends played a mediating role in this relationship. For the fear of being infected by the coronavirus and the stress affecting students’ academic performance, none of the strategies showed a complete mediating contribution to the relationship. Similar results were obtained for the mediating effect of the coping strategies for being worried, except for the role of optimism on the pandemic’s financial consequences relationship. In contrast, all coping strategies showed a mediating effect on the relationship between students’ financial concerns and depression. For the fear of being infected by the coronavirus, and the stress affecting student academic performance, none of the coping strategies played a complete mediating effect.

These results may suggest that optimism, emotional closeness, and gratitude allow young people to include in their cognitive bandwidth the new reality imposed by the pandemic, in particular, for the financial concerns related to the pandemic. Nevertheless, the mediating role of the coping strategies on the fear of being infected by coronavirus, and the stress affecting student academic performance, was partial or non-existent.

## 5. Discussion

In line with the available evidence regarding the consequences of the pandemic on students’ well-being and mental health [5,10,11,12,13], we found that the pandemic had negative consequences on student life, associated with their subjective well-being. The pandemic caused by COVID-19 affected students by reducing their life satisfaction and feelings of happiness, and by increasing depression and worry [69,70,71,72]. However, correlations between well-being variables were positive and positively correlated to gratitude, emotional closeness, and optimism. Similar to other studies conducted during the pandemic [48,49,59], we found that being optimistic and grateful helped students reduce the negative effect of fear of infection and the pandemic’s impact on students’ academic performance, after adjusting for age and gender. Results showed that students who are more optimistic, closer to their parents and friends, and grateful can cope better with a crisis. Likewise, these positive attitudes and emotions increased well-being and reduced the prevalence of depression and worry.

This study faces several limitations. An important one is the lack of measures of well-being and mental health before the quarantine unfolded, limiting the possibility of better capturing the effects attributed to the pandemic or other factors. Another limitation is the study’s cross-sectional nature, which only captured measures at one point during the pandemic. Likewise, this study does not represent the college population in the context studied. Lastly, the original survey did not include variables to control students’ socioeconomic conditions, which are pivotal in this crisis. Despite its limitations, this study aims to promoted a broader discussion of students’ well-being.

Results suggest that coping strategies are a pivotal component for overcoming difficult circumstances. In the case of optimism and gratitude, there is significant evidence showing their positive correlation with good mental health and well-being [30]. Students who practice gratitude have better results in achieving their goals, report less physical complaints, and are more optimistic [44]. Optimistic students have a higher probability of ending their studies than dropping out because they tend to adapt, expect better results, and confront problems better than pessimistic students [35]. There is also evidence that implementing gratitude-enhancing techniques in college can reduce dropout rates; one of the major concerns of this pandemic. Graduating from college gives students more opportunities in the long run, particularly in a middle-income country like Colombia. A spike in dropout rates not only harms students’ futures but also the accumulation of human capital in society. There is a need to foster gratitude interventions at the college level particularly, given the mounting evidence of its effectiveness for helping students coping with difiicult situations [49,58,73,74].

Gratitude, optimism, and happiness levels among college students positively influence their academic performance, college adaptation, and retention. The challenge is to implement interventions effectively. College programs can significantly contribute to issues such as students’ adaptation and the promotion of students’ activities to enhance their roles beyond academic responsibilities. Online interventions have been an option for college students who do not seek formal help [75,76,77]. Moreover, fostering interventions to promote gratitude, optimism or emotional closeness can reduce the financial concerns raised by the pandemic and allow students to manage their emotions and their expectations for the future.

## 6. Conclusions

This paper explored the mediating effects of optimism, emotional closeness, and gratitude on well-being during the pandemic caused by COVID-19. This analysis used information from an online survey collected in Cali, Colombia, among college students after one month of quarantine. Similar to other studies conducted with the same population, we found a negative impact of the pandemic on students’ well-being. Our results also showed that coping strategies such as optimism, gratitude, and emotional closeness reduced the negative consequences of a crisis. An important contribution of our study is contributing to the ongoing discussion of college students’ well-being by providing evidence from a context with little available data.

This study also provides arguments for the call to universities and the educational system to foster positive interventions, such as optimism and gratitude, by offering and promoting programs within the academic curricula. A substantial body of research from positive psychology shows that attitudes such as those studied in this analysis can have positive returns. Aspects of how to implement those strategies or transmit them to the student population are beyond the scope of this analysis. Along with the benefits of the programs, it is important to promote social connectedness in students. These activities promote optimism and gratitude at the individual level and should also provide an opportunity for students to improve their social relationships with other students.

## Figures and Tables

**Table 1:** Cronbach’s Alpha correlation for gratitude and optimism.

Optimism		
Item	Obs	alpha
In uncertain times, I usually expect the best.	634	0.74
I’m always optimistic about my future.	634	0.72
Overall, I expect more good things to happen to me than bad.	634	0.78
Test scale		0.82
Gratitute		
Item	Obs	alpha
I have so much in life to be thankful for.	634	0.87
If I had to list everything that I felt grateful for, it would be a very long list	634	0.87
When I look at the world, I see much to be grateful for.	634	0.87
I am grateful to a wide variety of people.	634	0.89
Long amounts of time can go by before I feel grateful to something or someone	634	0.89
Test scale		0.90

**Table 2:** Variables correlations.

Variables	Life Satisfaction	Happiness	Worried	Depression	Optimism	Emotional Closeness with Parents	Emotional Closeness with Friends	Gratitude	Financial Consequences	Fear of Being Infected	Academic Stress
Life satisfaction	1										
Happiness	0.62	1.00									
Worried	−0.22	−0.34	1.00								
Depression	−0.45	−0.59	0.44	1.00							
Optimism	0.48	0.40	−0.11	−0.32	1.00						
Emotional closeness with parents	0.38	0.32	−0.07	−0.23	**0.33**	1.00					
Emotional closeness with friends	0.38	0.28	0.02	−0.17	**0.26**	**0.29**	1.00				
Gratitude	0.41	0.33	0.02	−0.20	**0.43**	**0.43**	**0.33**	1.00			
Financial consequences	−0.03	−0.09	0.14	0.11	0.03	0.09	0.08	0.16	1.00		
Fear of being infected	−0.11	−0.16	0.16	0.15	−0.08	−0.02	−0.10	−0.05	0.17	1.00	
Academic stress	−0.15	−0.17	0.29	0.25	−0.13	−0.06	−0.03	−0.02	0.16	0.05	1

**Table 3:** Mediate effect of coping strategic on life satisfaction and happiness.

Mediate Effect	Independent Variable	Financial Consequences		Health Consequences		Academic Consequences	
		**Outcome: Life Satisfaction**					
Mediate variable	Estimates	Indirect effect	CI	Indirect effect	CI	Indirect effect	CI
Optimism	Delta	0.038	[0.001, 0.074]	−0.040	[−0.076, −0.004]	-0.065	[−0.102, −0.028]
	Sobel	0.038	[0.001, 0.074]	−0.040	[−0.076, −0.004]	−0.065	[−0.102, −0.028]
	Monte Carlo	0.037	[0.003, 0.074]	−0.041	[−0.077, −0.006]	−0.066	[−0.103, −0.031]
	RIT	1.970	Full mediation	0.394	Full mediation	0.426	Partial mediation
	RID	2.031		0.651		0.743	
Gratitude	Delta	0.07	[0.036, 0.105]	−0.033	[−0.065, 0.000]	−0.02	[−0.052, 0.013]
	Sobel	0.07	[0.036, 0.105]	−0.033	[−0.065, 0.000]	−0.02	[−0.052, 0.013]
	Monte Carlo	0.07	[0.038, 0.106]	−0.033	[−0.066, −0.001]	−0.02	[−0.053, 0.012]
	RIT	3.684	Full mediation	0.320	Partial mediation	0.128	No mediation
	RID	1.373		0.471		0.147	
Emotional closeness to parents	Delta	0.041	[0.010, 0.071]	−0.012	[−0.041, 0.017]	−0.031	[−0.061, −0.002]
	Sobel	0.041	[0.010, 0.071]	−0.012	[−0.041, 0.017]	−0.031	[−0.061, −0.002]
	Monte Carlo	0.04	[0.012, 0.071]	−0.013	[−0.041, 0.017]	−0.032	[−0.062, −0.004]
	RIT	2.132	Full mediation	0.116	No mediation	0.206	Partial mediation
	RID	1.884		0.132		0.259	
Emotional closeness to friends	Delta	0.044	[0.013, 0.074]	−0.043	[−0.073, −0.013]	−0.017	[−0.047, 0.012]
	Sobel	0.044	[0.013, 0.074]	−0.043	[−0.073, −0.013]	−0.017	[−0.047, 0.012]
	Monte Carlo	0.043	[0.015, 0.075]	−0.044	[−0.075, −0.016]	−0.018	[−0.047, 0.011]
	RIT	2.297	Full mediation	0.423	Full mediation	0.114	No mediation
	RID	1.771		0.732		0.129	
		**Outcome: Happiness**					
Mediate variable	Estimates	Indirect effect	CI	Indirect effect	CI	Indirect effect	CI
Optimism	Delta	0.030	[0.000, 0.060]	−0.032	[−0.062, −0.003]	−0.053	[−0.083, −0.022]
	Sobel	0.030	[0.000, 0.060]	−0.032	[−0.062, −0.003]	−0.053	[−0.083, −0.022]
	Monte Carlo	0.030	[0.002, 0.060]	−0.033	[−0.063, −0.005]	−0.053	[−0.084, −0.025]
	RIT	1.126	Full mediation	0.239	Partial mediation	0.316	Partial mediation
	RID	0.53		0.314		0.462	
Gratitude	Delta	0.058	[0.029, 0.088]	−0.027	[−0.055, 0.000]	−0.016	[−0.044, 0.011]
	Sobel	0.058	[0.029, 0.088]	−0.027	[−0.055, 0.000]	−0.016	[−0.044, 0.011]
	Monte Carlo	0.058	[0.031, 0.090]	−0.028	[−0.056, −0.001]	−0.017	[−0.044, 0.010]
	RIT	2.169	Partial mediation	0.200	Partial mediation	0.098	No mediation
	RID	0.684		0.25		0.109	
Emotional closeness to parents	Delta	0.035	[0.008, 0.061]	−0.010	[−0.035, 0.015]	−0.027	[−0.052, −0.001]
	Sobel	0.035	[0.008, 0.061]	−0.010	[−0.035, 0.015]	−0.027	[−0.052, −0.001]
	Monte Carlo	0.034	[0.010, 0.062]	−0.011	[−0.036, 0.014]	−0.027	[−0.054, −0.003]
	RIT	1.288	Full mediation	0.075	No mediation	0.161	Partial mediation
	RID	0.563		0.081		0.192	
Emotional closeness to friends	Delta	0.031	[0.008, 0.054]	−0.030	[−0.052, −0.008]	−0.012	[−0.033, 0.009]
	Sobel	0.031	[0.009, 0.053]	−0.030	[−0.052, −0.008]	−0.012	[−0.033, 0.009]
	Monte Carlo	0.031	[0.010, 0.055]	−0.031	[−0.055, −0.011]	−0.013	[−0.035, 0.008]
	RIT	1.149	Full mediation	0.224	Partial mediation	0.074	No mediation
	RID	0.535		0.289		0.080	

Note: RIT = (Indirect effect/Total effect), RID = (Indirect effect/Direct effect), Delta = (a × b).

**Table 4:** Mediate effect of coping strategic on worry and depression.

Mediate Effect	Independent Variable	Financial Consequences		Health Consequences		Academic Consequences	
		**Outcome: Worry**					
Mediate variable	Estimates	Indirect effect	CI	Indirect effect	CI	Indirect effect	CI
Optimism	Delta	0.031	[0.008, 0.054]	−0.030	[−0.052, −0.008]	−0.012	[−0.033, 0.009]
	Sobel	0.031	[0.009, 0.053]	−0.030	[−0.052, −0.008]	−0.012	[−0.033, 0.009]
	Monte Carlo	0.031	[0.010, 0.055]	−0.031	[−0.055, −0.011]	−0.013	[−0.035, 0.008]
	RIT	1.149	Full mediation	0.224	Partial mediation	0.074	No mediation
	RID	0.535		0.289		0.080	
Gratitude	Delta	0.004	[−0.008, 0.017]	−0.002	[−0.008, 0.004]	−0.001	[−0.005, 0.003]
	Sobel	0.004	[−0.008, 0.017]	−0.002	[−0.008, 0.004]	−0.001	[−0.005, 0.003]
	Monte Carlo	0.005	[−0.008, 0.019]	−0.002	[−0.011, 0.004]	−0.001	[−0.008, 0.003]
	RIT	0.067	No mediation	0.015	No mediation	0.004	No mediation
	RID	0.072		0.015		0.004	
Emotional closeness to parents	Delta	−0.006	[−0.015, 0.003]	0.002	[0.003, 0.007]	0.005	[−0.003, 0.012]
	Sobel	−0.006	[−0.015, 0.003]	0.002	[0.003, 0.007]	0.005	[−0.003, 0.012]
	Monte Carlo	−0.006	[−0.016, 0.002]	0.002	[0.003, 0.009]	0.005	[−0.002, 0.015]
	RIT	0.094	No mediation	0.013	No mediation	0.017	No mediation
	RID	0.086		0.013		0.018	
Emotional closeness to friends	Delta	0.004	[−0.005, 0.013]	−0.004	[−0.013, 0.005]	−0.002	[−0.006, 0.003]
	Sobel	0.004	[−0.005, 0.013]	−0.004	[−0.013, 0.005]	−0.002	[−0.006, 0.003]
	Monte Carlo	0.004	[−0.005, 0.015]	−0.004	[−0.016, 0.005]	−0.002	[−0.009, 0.003]
	RIT	0.061	No mediation	0.029	No mediation	0.006	No mediation
	RID	0.065		0.028		0.006	
		**Outcome: Depression**					
Mediate variable	Estimates	Indirect effect	CI	Indirect effect	CI	Indirect effect	CI
Optimism	Delta	−0.022	[−0.044, 0.000]	0.024	[0.002, 0.045]	0.038	[0.015, 0.061]
	Sobel	−0.022	[−0.044, 0.000]	0.024	[0.002, 0.045]	0.038	[0.015, 0.061]
	Monte Carlo	−0.021	[−0.043, −0.002]	0.024	[0.003, 0.046]	0.038	[0.017, 0.063]
	RIT	0.746	Partial mediation	0.192	Partial mediation	0.155	Partial mediation
	RID	0.427		0.237		0.184	
Gratitude	Sobel	−0.036	[−0.007, 0.027]	0.017	[−0.001, 0.034]	0.010	[−0.056, −0.015]
	Monte Carlo	−0.035	[−0.006, 0.028]	0.017	[0.001, 0.036]	0.010	[−0.056, −0.017]
	RIT	1.203	Full mediation	0.134	Partial mediation	0.04	No mediation
	RID	0.546		0.155		0.042	
Emotional closeness to parents	Delta	−0.024	[−0.044, −0.005]	0.007	[−0.010, 0.025]	0.019	[0.000, 0.037]
	Sobel	−0.024	[−0.044, −0.005]	0.007	[−0.010, 0.025]	0.019	[0.000, 0.037]
	Monte Carlo	−0.024	[−0.043, −0.007]	0.007	[−0.010, 0.025]	0.019	[0.002, 0.039]
	RIT	0.827	Full mediation	0.058	No mediation	0.077	Partial mediation
	RID	0.453		0.062		0.083	
Emotional closeness to friends	Delta	−0.017	[−0.031, −0.003]	0.017	[0.003, 0.031]	0.007	[−0.005, 0.018]
	Sobel	−0.017	[−0.031, −0.003]	0.017	[0.003, 0.031]	0.007	[−0.005, 0.018]
	Monte Carlo	−0.016	[−0.032, −0.004]	0.017	[0.004, 0.033]	0.007	[−0.004, 0.020]
	RIT	0.571	Full mediation	0.135	Partial mediation	0.027	No mediation
	RID	0.364		0.156		0.028	

Note: RIT = (Indirect effect/Total effect), RID = (Indirect effect/Direct effect), Delta = (Beta × Alpha).

## Data Availability

Available data for this study can be accessed at Martínez, L., Valencia, I., & Trofimoff, V. (2020). Subjective wellbeing and mental health during the COVID-19 pandemic: Data from three population groups in Colombia. Data in brief, 32, 106287.

## Appendix A

			STUDENT WELL-BEING DURING THE PANDEMIC																
Date:		Month		Day		Year													
1. During the process of collecting, typing and manipulating the data provided by you. These data will be used only for academic purposes and will be presented in an aggregated way. Given the above, are you willing to participate in this survey?									3. How old are you?										
1	Yes				0		No		4. What is your academic program?										

									5. What semester are you in?										
6. Gender																			
	1	Male											2		Female				
SUBJECTIVE WELL-BEING																			
The next question is about how satisfied you are, on a scale of 0 to 10. Zero means that you are not at all satisfied and 10 means that you are completely satisfied.																			
7. In general, how satisfied are you with all aspects of your life?																			
Not at all satisfied			0	1	2	3	4	5	6	7	8	9	10	Completely satisfied					
The next questions are about how you felt yesterday on a scale of 0 to 10. Zero means that you did not experience these feelings “at any time” while 10 means that you experienced these feelings “all the time.” Now I am going to read you a list of scenarios that you could experience																			
At any time			0	1	2	3	4	5	6	7	8	9	10	All the time					
8. How happy?			0	1	2	3	4	5	6	7	8	9	10						
9. How worried?			0	1	2	3	4	5	6	7	8	9	10						
10. How depressed?			0	1	2	3	4	5	6	7	8	9	10						
CORONAVIRUS AND WELL-BEING																			
In the next questions, be as honest as possible and try not to let your answer to the first statement of each question influence the answers to the other statements.																			
11. Please answer how strongly you agree or disagree with the following statements regarding Coronavirus.																			
Completely disagree			0	1	2	3	4	5	6	7	8	9	10	Completely agree					
11.1	I am concerned about my health.							0	1	2	3	4	5	6	7	8	9	10	
11.2	I am concerned about the health of my loved ones.							0	1	2	3	4	5	6	7	8	9	10	
11.3	I am taking all the protective measures recommended by the media and the government.							0	1	2	3	4	5	6	7	8	9	10	
11.4	With government measures, I feel isolated.							0	1	2	3	4	5	6	7	8	9	10	
11.5	I consider that the quarantine is an individual							0	1	2	3	4	5	6	7	8	9	10	
	responsibility and not of the government.																		
11.6	I enjoy having time to spend with my family							0	1	2	3	4	5	6	7	8	9	10	
11.7	I enjoy being able to disconnect from my daily activities							0	1	2	3	4	5	6	7	8	9	10	
11.8	I feel more productive working at home or							0	1	2	3	4	5	6	7	8	9	10	
	independently.																		
11.9	I keep informed and read the news about							0	1	2	3	4	5	6	7	8	9	10	
	the Coronavirus.																		
11.10	I am concerned about the financial consequences							0	1	2	3	4	5	6	7	8	9	10	
	of the Coronavirus.																		
11.11	The probability that my loved ones or I getting infected is high.							0	1	2	3	4	5	6	7	8	9	10	
11.12	I feel that in last days my anxiety and stress levels							0	1	2	3	4	5	6	7	8	9	10	
	have increased.																		
11.13	I consider that the government is taking all the necessary measures to overcome the crisis.							0	1	2	3	4	5	6	7	8	9	10	
11.14	I consider that the government provides sufficient information in these cases.							0	1	2	3	4	5	6	7	8	9	10	
11.15	I’m amused by memes and jokes about the Coronavirus.							0	1	2	3	4	5	6	7	8	9	10	
11.16	I feel that with the help of technology I am ready to continue my activities from home.							0	1	2	3	4	5	6	7	8	9	10	
11.17	I always check the sources of information to be reliable, before commenting or sharing the news.							0	1	2	3	4	5	6	7	8	9	10	
11.18	I am concerned that my academic performance will be affected by the Coronavirus.							0	1	2	3	4	5	6	7	8	9	10	
11.19	I am comfortable with the online classes.							0	1	2	3	4	5	6	7	8	9	10	
1																			

		STUDENT WELL-BEING DURING THE PANDEMIC																
MANAGING EMOTIONS																		
12. Please answer the following questions:																		
Completely disagree		0	1	2	3	4	5	6	7	8	9	10	Completely agree					
12.1 In difficult times I usually hope for the best					0	1	2	3	4	5	6	7	8	9	10			
12.2 I getting relax easily					0	1	2	3	4	5	6	7	8	9	10			
12.3 I am always optimistic about the future					0	1	2	3	4	5	6	7	8	9	10			
12.4 I really enjoy hangout with my friends					0	1	2	3	4	5	6	7	8	9	10			
12.5 It is important to me always be busy					0	1	2	3	4	5	6	7	8	9	10			
12.6 I don’t get upset easily					0	1	2	3	4	5	6	7	8	9	10			
12.7 Overall, I hope more good things than bad things happen to me					0	1	2	3	4	5	6	7	8	9	10			
13. How happy are you with:																		
Unhappy		0	1	2	3	4	5	6	7	8	9	10	Happy					
13.1	Your life overall						0	1	2	3	4	5	6	7	8	9	10	
13.2	At this moment in your life						0	1	2	3	4	5	6	7	8	9	10	
13.3	Yourself						0	1	2	3	4	5	6	7	8	9	10	
13.4	Your physical appearance						0	1	2	3	4	5	6	7	8	9	10	
13.5	Your ability to communicate with others						0	1	2	3	4	5	6	7	8	9	10	
13.6	Your health overall						0	1	2	3	4	5	6	7	8	9	10	
13.7	What you have achieved in your life so far						0	1	2	3	4	5	6	7	8	9	10	
13.8	The college						0	1	2	3	4	5	6	7	8	9	10	
13.9	Your college classmates						0	1	2	3	4	5	6	7	8	9	10	
14. How stressed are you with:																		
Not stressed at all		0	1	2	3	4	5	6	7	8	9	10	Very stressed					
14.1	Your life overall						0	1	2	3	4	5	6	7	8	9	10	
14.2	The college						0	1	2	3	4	5	6	7	8	9	10	
14.3	Your home						0	1	2	3	4	5	6	7	8	9	10	
14.4	The financial situation of your home						0	1	2	3	4	5	6	7	8	9	10	
14.5	The lack of time						0	1	2	3	4	5	6	7	8	9	10	
14.6	Your future						0	1	2	3	4	5	6	7	8	9	10	
15. How often do you use stress management techniques:																		
At any time		0	1	2	3	4	5	6	7	8	9	10	All the time					
15.1	Breathe deeply						0	1	2	3	4	5	6	7	8	9	10	
15.2	Count to ten						0	1	2	3	4	5	6	7	8	9	10	
15.3	Praying						0	1	2	3	4	5	6	7	8	9	10	
15.4	Meditating						0	1	2	3	4	5	6	7	8	9	10	
15.5	Listen to music						0	1	2	3	4	5	6	7	8	9	10	
15.6	Doing exercise						0	1	2	3	4	5	6	7	8	9	10	
15.7	Stretching exercises						0	1	2	3	4	5	6	7	8	9	10	
15.8	Talking or calling someone						0	1	2	3	4	5	6	7	8	9	10	
15.9	Imagine something pleasant						0	1	2	3	4	5	6	7	8	9	10	
15.10	Look at the big picture of the problem						0	1	2	3	4	5	6	7	8	9	10	
15.11	Writing down the factors that stress me						0	1	2	3	4	5	6	7	8	9	10	
15.12	Thank everyday						0	1	2	3	4	5	6	7	8	9	10	
16. How emotionally close do you feel to your loved ones:																		
Not close at all		0	1	2	3	4	5	6	7	8	9	10	Completely close					
16.1 Parents		0	1	2	3	4	5	6	7	8	9	10						
16.2 Friends		0	1	2	3	4	5	6	7	8	9	10						
16.3 Couple		0	1	2	3	4	5	6	7	8	9	10						
16.4 College classmates		0	1	2	3	4	5	6	7	8	9	10						
TWith the following question, we want to know how much you usually thank and appreciate certain everyday situations or your life.																		
17. How much do you agree with?																		
Completely disagree		0	1	2	3	4	5	6	7	8	9	10	Completely agree					
17.1	I have a lot to thank life						0	1	2	3	4	5	6	7	8	9	10	
17.2	If I had to make a gratitude list, it would be very long						0	1	2	3	4	5	6	7	8	9	10	
17.3	When I look at the world I have a lot to thank						0	1	2	3	4	5	6	7	8	9	10	
17.4	I am grateful with a lot of people						0	1	2	3	4	5	6	7	8	9	10	
17.5	As time goes by, I appreciate more the people, events and situations that are part of my life.						0	1	2	3	4	5	6	7	8	9	10	
2																		
